# Conventional and novel hypoxemia-related indices for obstructive sleep apnea severity and incident hypertension: a prospective cohort study

**DOI:** 10.3389/fneur.2026.1973505

**Published:** 2026-10-07

**Authors:** Lixia Wang, Xinjie Hui, Rong Huang, Yi Xiao

**Affiliations:** 1 Department of Pathology, West China Hospital, Sichuan University, Chengdu, China; 2 Department of Pulmonary and Critical Care Medicine, Peking Union Medical College Hospital, Chinese Academy of Medical Sciences & Peking Union Medical College, Beijing, China; 3 Department of Pulmonary and Critical Care Medicine, Shaanxi Provincial People’s Hospital, Xi'an, China

**Keywords:** apnea–hypopnea index, hypertension, hypoxic burden, obstructive sleep apnea, sleep breathing impairment index

## Abstract

**Background and objective:**

Obstructive sleep apnea (OSA) is associated with hypertension (HTN), but the apnea–hypopnea index (AHI) and oxygen desaturation index (ODI) may not fully characterize nocturnal hypoxemia relevant to HTN development. We evaluated the associations of the sleep breathing impairment index (SBII) and hypoxic burden (HB) with incident HTN and compared their discriminative performance with AHI and ODI.

**Methods:**

This prospective study included 601 participants with OSA (AHI ≥ 15 events/h) from Sleep Heart Health Study who were free of HTN at baseline. SBII, HB, AHI and ODI were analyzed both in quartiles and as continuous variables. Multivariable logistic regression and restricted cubic spline analyses were used to evaluate their associations with incident HTN. Discrimination performance was assessed by comparing a clinical base model with models that separately incorporating each quartile-categorized index.

**Results:**

During a median follow-up of approximately 5 years, 194 (32.3%) participants developed HTN. In the primary fully adjusted model, the highest quartiles of SBII (OR, 1.71; 95% CI, 1.02–2.90; *p* = 0.044) and HB (OR, 1.69; 95% CI, 1.02–2.79; *p* = 0.041) were associated with higher odds of incident HTN. In the T90-adjusted sensitivity model, both associations were significant, whereas AHI and ODI quartiles remained nonsignificant. The clinical base model had a C-statistic of 0.614; adding SBII or HB, did not significantly improve discrimination.

**Conclusion:**

Exploratory quartile-based analyses suggested that the highest quartiles of SBII and HB were associated with higher odds of incident HTN among participants with OSA, whereas AHI and ODI were not. These findings indicate that marked elevation in SBII and HB may provide a more comprehensive characterization of OSA-related nocturnal hypoxemia relevant to incident HTN. However, the nonsignificant continuous and overall spline analyses and the limited incremental discrimination warrant cautious interpretation and further validation.

## Introduction

Obstructive sleep apnea (OSA) is a common sleep-related breathing disorder characterized by recurrent upper-airway obstruction, intermittent hypoxemia, and sleep fragmentation. It is estimated that nearly one billion adults worldwide have OSA, highlighting its substantial public health burden (1). Hypertension (HTN) is among the most common cardiovascular comorbidities of OSA and represents an important pathway through which OSA may contribute to cardiovascular morbidity and mortality (2, 3). OSA-related mechanisms such as repeated hypoxia and reoxygenation, arousal responses, and marked negative intrathoracic pressure can contribute to persistent increases in blood pressure (BP) by activating the sympathetic nervous system, enhancing oxidative stress, provoking systemic inflammation, impairing endothelial function, and disturbing renin–angiotensin–aldosterone system regulation (2, 4). However, considerable heterogeneity exists in the cardiovascular comorbidities of OSA, suggesting that conventional measures may not adequately identify patients who are particularly susceptible to HTN.

The apnea–hypopnea index (AHI) and oxygen desaturation index (ODI) are commonly used to characterize OSA severity. AHI quantifies the frequency of apneas and hypopneas, whereas ODI measures the frequency of oxygen desaturations exceeding a predefined threshold. Although clinically useful, neither metric fully captures the depth, duration, and cumulative magnitude of respiratory event-related hypoxemia (5–7). Moreover, previous studies have shown that AHI and ODI are not significantly associated with reductions in BP, nor with the circadian systolic BP rhythm or nocturnal HTN (6, 7). Therefore, these frequency-based indices may incompletely characterize the nocturnal hypoxic burden relevant to BP dysregulation.

In recent years, integrated measures such as hypoxic burden (HB) and the sleep breathing impairment index (SBII) have been developed to provide a more comprehensive assessment of OSA-related physiological burden. Both indices incorporate information on respiratory event-associated oxygen desaturation beyond event frequency alone, thereby capturing the cumulative magnitude of nocturnal hypoxic exposure (8–10). Previous studies have linked higher HB and SBII levels to adverse cardiovascular outcomes; however, evidence comparing their associations with incident HTN against those of conventional frequency-based indices remains limited (6, 7, 9, 10).

Using data from the Sleep Heart Health Study (SHHS), we investigated the prospective associations of SBII, HB, ODI, and AHI with incident HTN among participants with OSA and without HTN at baseline. We further explored their incremental discriminative performance by comparing a clinical base model with models separately incorporating SBII, HB, AHI, or ODI. This study aimed to clarify the potential relevance of integrated hypoxemia indices to incident HTN and compare their performance with conventional frequency-based measures in a community-based cohort.

## Study design and methods

### Participants

Data for the present analysis were obtained from the Sleep Heart Health Study (SHHS, https://sleepdata.org/datasets/shhs/), a community-based, multicenter, prospective cohort established to evaluate the cardiovascular consequences of sleep-disordered breathing (11, 12). The baseline examination (SHHS examination 1) was conducted between 1995 and 1998 and enrolled 6,441 middle-aged and older adults. At enrollment, participants completed standardized questionnaires assessing sociodemographic characteristics, anthropometric and BP measurements, and underwent unattended overnight polysomnography (PSG). Participants underwent a second examination between 2001 and 2003, after a median follow-up of approximately 5 years, at which time BP was reassessed. Ultimately, 601 participants with OSA and without pre-existing HTN were included in the present study (Figure 1). All participants signed informed consent, and institutional review board approval was obtained from each participating institution.

**Figure 1:**
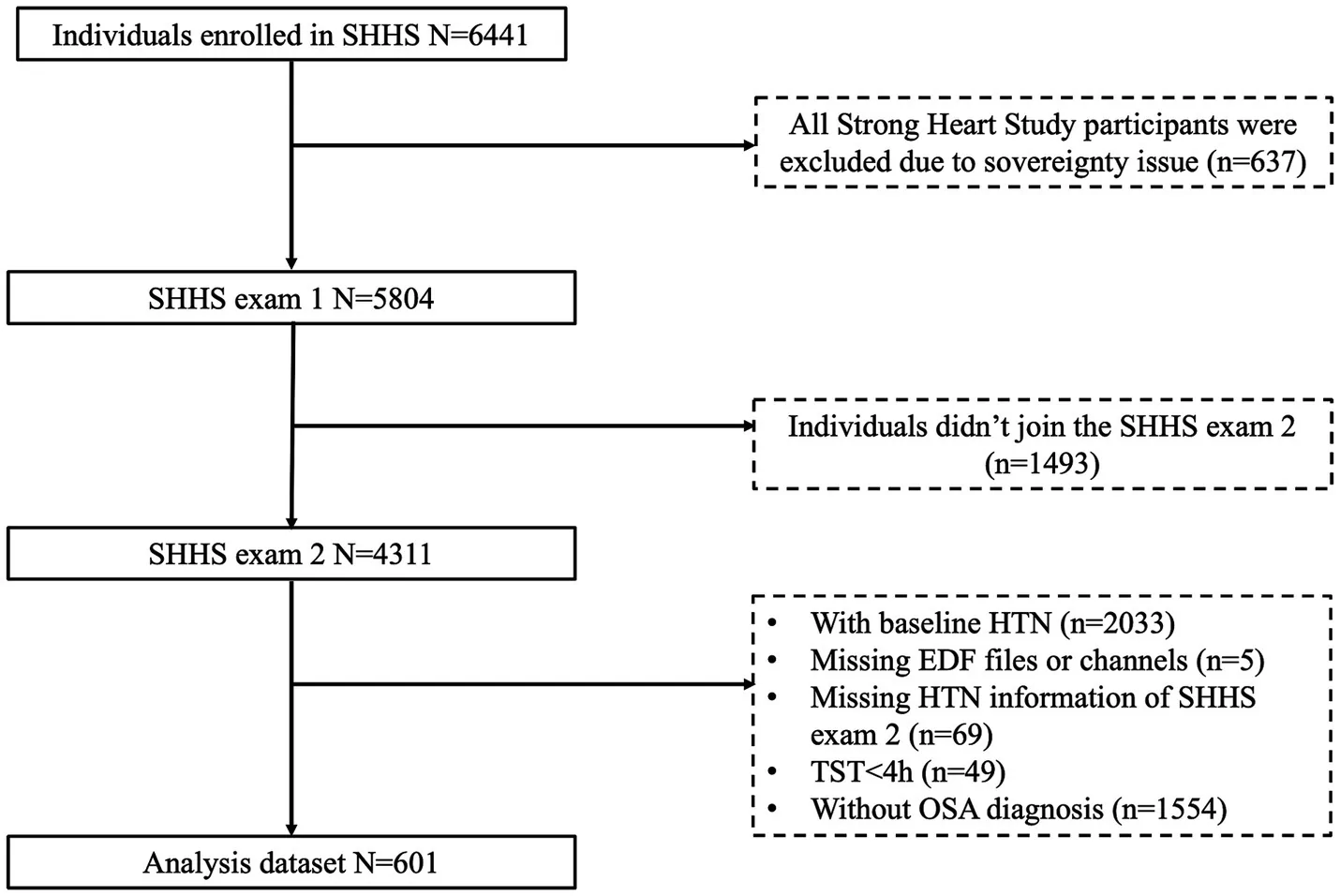
Flow chart of this study. SHHS, the Sleep Heart Health Study; HTN, hypertension; EDF, European data format; TST, total sleep time.

### Events definition

At both SHHS examinations, participants rested in a seated position for 5 min before three consecutive manual BP measurements were obtained. The mean of the second and third readings was used in the analyses. HTN was defined by the presence of any of the following: systolic BP ≥ 140 mmHg, diastolic BP ≥ 90 mmHg, or current use of antihypertensive medication. We also collected demographic characteristics, alcohol consumption, smoking history, medical history [such as history of diabetes mellitus (DM), and chronic obstructive pulmonary disease], medication use within 2 weeks prior to enrollment.

OSA was defined by an AHI ≥ 15 events/h during sleep (13). AHI was calculated as the count of apneas and hypopneas with a flow reduction of ≥30% and oxygen desaturation of ≥3%, with or without arousal, per hour of sleep. Body mass index (BMI) was computed by dividing weight by the square of height (kg/m^2^). The percentage of sleep time spent with oxygen saturation of <90% (T90) was used to describe nocturnal hypoxemia.

### Calculation of SBII and HB

Continuous SpO₂ signals sampled at 1 Hz and manually verified respiratory-event annotations were extracted from the PSG-EDF files and corresponding XML files, respectively, and used to calculate both SBII and HB. SBII was calculated using methods previously reported by our team (9, 10). For each apnea or hypopnea, an associated SpO₂ desaturation of ≥3% beginning within 100 s of event onset was identified. The corresponding desaturation area, including the decline and recovery phases, was multiplied by the event duration. These event-specific products were summed and divided by total sleep time, yielding SBII in %min^2^/h (details of SBII algorithm included in Online supplement). HB was calculated in this cohort following the previous study, capturing the cumulative area of event-related desaturations and expressing as %min/h (8).

### Statistical analysis

Continuous variables were reported as mean ± standard deviation or median [P50 (P25, P75)], as appropriate. Categorical variables were presented as numbers and percentages [*n* (%)]. Because incident HTN was ascertained at the SHHS2 examination and the exact date of HTN onset was unavailable, multivariate logistic regression analysis was conducted to assess the associations of SBII, HB, AHI, and ODI with incident HTN. Each index was analyzed both continuously and in quartiles based on its distribution in the analytic sample. Covariates included in the primary models were selected *a priori* based on clinical relevance and their plausible roles as confounders of the associations between OSA-related hypoxemia and incident HTN. Baseline systolic and diastolic BP were not included in the primary models because subthreshold BP elevations may partly reflect prior effects of chronic OSA-related hypoxemia; adjusting for them could therefore attenuate the overall prospective associations of interest.

Model 0 was unadjusted. In Model 1, we adjusted for gender, age [binary covariate (<60 versus ≥60 years); previous study indicated that age ≥60 years was association with incident HTN (14)], race, BMI [BMI classifications encompassed non-obese (BMI < 30 kg/m^2^), obese class I (BMI 30–35 kg/m^2^), beyond class II obesity (BMI ≥ 35 kg/m^2^); previous population-based and prospective studies showing progressively greater HTN prevalence across the increasing BMI categories (15)], current smoking, alcohol use. In Model 2, we adjusted for coexisting DM and chronic obstructive pulmonary disease, plus covariates in Model 1. Due to T90 represents a competing measure of nocturnal hypoxemia, it was not included in the primary adjustment set. In sensitivity model, we adjusted for T90 as a binary covariate (<5% versus ≥5%), plus covariates included in Model 2; the cutoff was based on previous evidence of its associated with HTN in OSA (16). Missing covariate data were handled using complete-case analysis, and no imputation was performed (details are shown in Supplementary Table E1).

Potential nonlinear associations between each continuous OSA severity metric and incident HTN were evaluated using restricted cubic spline with three knots placed at the 10th, 50th, and 90th percentiles in fully adjusted logistic regression model. The median was used as the reference. All exposure values were retained without truncation or winsorization, while the curves were displayed from the 1st to the 99th percentile with rug plots indicating data density. Overall and nonlinear associations were assessed using Wald chi-square tests.

The discriminative ability of SBII, HB, AHI, and ODI for incident HTN was quantified using the C-statistic. Pairwise differences in C-statistics were evaluated using the DeLong test. All analyses were performed using R version 4.2.1,^1^ with a two-tailed *p* value < 0.05 defining statistical significance.

## Results

### Baseline characteristics

This study included 601 participants with OSA and without pre-existing HTN. During a median follow-up of approximately 5 years, 194 participants (32.3%) developed HTN. At baseline, the median age was 62.0 years, the median BMI was 29.2 kg/m^2^, and 65.4% of participants were male. The cohort was predominantly White (91.2%), followed by Black or African American (3.2%) and other racial groups (5.7%). Compared with excluded participants, those included in the analytic sample were less frequently male, more frequently White, had higher BMI, lower baseline BP, and more severe sleep apnea and hypoxemia (Supplementary Table E2). Baseline characteristics of analytic sample according to incident HTN status were summarized in Table 1. Compared with those without incident HTN, participants who developed HTN were older, and had higher BMI and baseline systolic BP. They also more pronounced nocturnal hypoxemia, as reflected by higher T90 and HB.

**Table 1:** Baseline characteristics between participants with and without incident HTN.

Variable	Total (*N* = 601)	No incident HTN (*N* = 407)	Incident HTN (*N* = 194)	*p*
Age, years	62.00 (56.00, 70.00)	62.00 (55.00, 70.00)	64.50 (58.00, 73.00)	0.003
Age group				0.032
<60 years (*n* = 221)	221 (36.77)	162 (39.80)	59 (30.41)	
⩾60 years (*n* = 380)	380 (63.23)	245 (60.20)	135 (69.59)	
Male, *n* (%)	393 (65.39)	262 (64.37)	131 (67.53)	0.504
BMI, kg/m^2^	29.21 (26.26, 31.98)	28.80 (25.97, 31.57)	29.83 (27.27, 33.07)	0.004
BMI group				0.020
<30 kg/m^2^ (*n* = 352)	352 (58.57)	252 (61.92)	100 (51.55)	
30 ~ 35 kg/m^2^ (*n* = 179)	179 (29.78)	115 (28.26)	64 (32.99)	
⩾35 kg/m^2^ (*n* = 70)	70 (11.65)	40 (9.83)	30 (15.46)	
Race, *n* (%)				0.174
White	548 (91.18)	377 (92.63)	171 (88.14)	
African American	19 (3.16)	10 (2.46)	9 (4.64)	
Other	34 (5.66)	20 (4.91)	14 (7.22)	
Alcohol use, *n* (%)	288 (51.34)	187 (49.60)	101 (54.89)	0.277
Current smoking, *n* (%)	52 (8.67)	36 (8.87)	16 (8.25)	0.815
Average systolic BP, mm Hg	118.00 (110.00, 127.00)	116.00 (108.00, 123.75)	124.00 (116.00, 131.00)	<0.001
Average diastolic BP, mm Hg	70.00 (64.00, 76.50)	70.00 (64.00, 76.00)	72.00 (66.00, 78.00)	0.056
DM, *n* (%)	25 (4.28)	15 (3.78)	10 (5.35)	0.512
COPD, *n* (%)	3 (0.50)	3 (0.74)	0 (0.00)	0.171
Antidepressants usage, *n* (%)	26 (4.35)	19 (4.69)	7 (3.63)	0.702
Benzodiazepines usage, *n* (%)	18 (3.01)	9 (2.22)	9 (4.66)	0.168
Follow-up duration, years	5.23 (5.08, 5.38)	5.23 (5.10, 5.39)	5.17 (5.02, 5.37)	0.014
AHI, events/h	24.05 (18.15, 33.72)	23.88 (17.97, 32.33)	24.33 (18.49, 35.47)	0.216
ODI, events/h	17.46 (12.43, 25.37)	17.44 (12.75, 24.77)	17.75 (11.79, 26.34)	0.607
SBII, %min2/h	23.95 (15.16, 39.77)	23.50 (14.59, 38.53)	24.78 (16.87, 44.69)	0.159
HB, %min/h	62.90 (40.67, 96.33)	59.10 (39.14, 92.45)	67.56 (44.04, 109.33)	0.045
T90, %	1.70 (0.37, 5.47)	1.44 (0.34, 4.84)	2.51 (0.45, 6.53)	0.022

Values were presented as median (interquartile range) or number (percentage).

HTN, hypertension; BMI, body mass index; SBP, systolic blood pressure; DBP, diastolic blood pressure; DM, diabetes mellitus; COPD, chronic obstructive pulmonary disease; SBII, sleep breathing impairment index; HB, hypoxic burden; AHI, apnea-hypopnea index; ODI, oxygen desaturation index; T90, the percentage of sleep time spent with oxygen saturation of <90%.

The incidence proportions of HTN according to SBII, HB, AHI and ODI quartiles were presented in Supplementary Table E3. A significant overall difference was observed across AHI quartiles (Global *p* = 0.015), whereas a significant increasing trend was observed across HB quartiles (*P* for trend = 0.023). No significant overall differences or linear trends were observed for SBII or ODI.

Pearson correlation analysis revealed significant positive correlations among SBII, HB, AHI, and ODI, with correlation coefficients ranging from 0.532 to 0.909 (all *p* < 0.001; Table 2 and Supplementary Figure E1). The strongest correlation was observed between SBII and HB (*r* = 0.909).

**Table 2:** Pearson correlations among SBII, HB, AHI, and ODI.

Variables	SBII	HB	AHI	ODI
SBII	—	0.909***	0.532***	0.626***
HB	0.909***	—	0.544***	0.649***
AHI	0.532***	0.544***	—	0.686***
ODI	0.626***	0.649***	0.686***	—

^***^p < 0.001.

SBII, sleep breathing impairment index; HB, hypoxic burden; AHI, apnea-hypopnea index; ODI, oxygen desaturation index.

### Association of SBII, HB, AHI, and ODI with incident HTN

Table 3 summarized the associations of SBII, HB, AHI, and ODI with incident HTN. In the unadjusted model (Model 0), the fourth quartile of SBII (OR, 1.64, 95% CI, 1.01–2.67; *p* = 0.046), HB (OR, 1.67, 95% CI, 1.03–2.70; *p* = 0.036) and AHI (OR, 1.83, 95% CI, 1.13–2.97; *p* = 0.014) were associated with higher odds of incident HTN than those in the first quartile, respectively. After multivariable adjustment in Model 2, the highest quartiles of SBII and HB remained associated with higher odds of incident HTN, with ORs of 1.71 (95% CI, 1.02–2.90; *p* = 0.044) and 1.69 (95% CI, 1.02–2.79; *p* = 0.041), respectively. In contrast, none of the AHI or ODI quartiles were significantly associated with incident HTN. When modeled continuously, none of the four indices showed a significant association per 1-SD increase in Model 2 (all *p* > 0.05).

**Table 3:** Association of SBII, HB, AHI, and ODI with incident HTN.

Variables	Model 0 OR (95%CI)	*p*	Model 1 OR (95%CI)	*p*	Model 2 OR (95%CI)	*p*
SBII, %min^2^/h						
Per 1-SD increase	1.06 (0.90–1.27)	0.479	1.04 (0.87–1.24)	0.665	1.04 (0.87–1.24)	0.672
Q1 (<15.16)	1.00	—	1.00	—	1.00	—
Q2 (≥15.16 and ≤23.95)	1.30 (0.79–2.13)	0.299	1.32 (0.79–2.23)	0.291	1.33 (0.79–2.25)	0.293
Q3 (>23.95 and ≤39.77)	1.08 (0.65–1.78)	0.771	1.11 (0.65–1.90)	0.709	1.10 (0.64–1.89)	0.724
Q4 (>39.77)	1.64 (1.01–2.67)	0.046	1.67 (0.99–2.82)	0.054	1.71 (1.02–2.90)	0.044
HB, %min/h						
Per 1-SD increase	1.13 (0.95–1.34)	0.161	1.10 (0.92–1.31)	0.315	1.09 (0.91–1.31)	0.330
Q1 (<40.66)	1.00	—	1.00	—	1.00	—
Q2 (≥40.66 and ≤62.90)	0.95 (0.57–1.56)	0.827	1.07 (0.63–1.82)	0.813	0.99 (0.57–1.70)	0.964
Q3 (>62.90 and ≤96.33)	1.11 (0.68–1.82)	0.679	1.09 (0.64–1.85)	0.750	1.07 (0.63–1.83)	0.796
Q4 (>96.33)	1.67 (1.03–2.70)	0.036	1.66 (0.99–2.77)	0.053	1.69 (1.02–2.79)	0.041
AHI, events/h						
Per 1-SD increase	1.18 (0.99–1.40)	0.059	1.11 (0.93–1.33)	0.255	1.11 (0.93–1.33)	0.263
Q1 (<18.15)	1.00	—	1.00	—	1.00	—
Q2 (≥18.15 and ≤24.05)	1.38 (0.85–2.25)	0.200	1.38 (0.82–2.31)	0.224	1.33 (0.79–2.24)	0.284
Q3 (>24.05 and ≤33.72)	0.88 (0.53–1.47)	0.626	0.84 (0.49–1.43)	0.532	0.81 (0.48–1.39)	0.453
Q4 (>33.72)	1.83 (1.13–2.97)	0.014	1.51 (0.90–2.54)	0.117	1.49 (0.89–2.51)	0.132
ODI, events/h						
Per 1-SD increase	1.13 (0.95–1.35)	0.160	1.11 (0.93–1.34)	0.24	1.11 (0.93–1.33)	0.246
Q1 (<12.43)	1.00	—	1.00	—	1.00	—
Q2 (≥12.43 and ≤17.46)	0.62 (0.38–1.00)	0.053	0.79 (0.47–1.33)	0.368	0.79 (0.47–1.32)	0.368
Q3 (>17.46 and ≤25.37)	0.68 (0.42–1.10)	0.121	0.79 (0.47–1.31)	0.355	0.73 (0.43–1.22)	0.227
Q4 (>25.37)	0.98 (0.61–1.57)	0.940	1.07 (0.65–1.76)	0.796	1.05 (0.63–1.74)	0.854

Data were presented as odds ratios (95% CIs). Model 0 was unadjusted; Model 1 was adjusted for age, race, gender, current smoking, alcohol use, and body mass index; Model 2 was adjusted for diabetes mellitus and chronic obstructive pulmonary disease plus covariates in Model 1.

Model 0 included 601 participants (194 incident HTN events), Model 1 included 560 participants (184 incident HTN events), and Model 2 included 557 participants (181 incident HTN events).

SBII, the sleep breathing impairment index; HB, hypoxic burden; ODI, oxygen desaturation index; AHI, apnea-hypopnea index; HTN, hypertension; CI, confidence interval, OR, odds ratio.

Additional adjustment for T90 did not materially alter the findings (Supplementary Table E4). The highest quartiles of SBII (OR, 1.80; 95% CI, 1.04–3.12; *p* = 0.036) and HB (OR, 1.80; 95% CI, 1.05–3.10; *p* = 0.034) remained associated with higher odds of incident HTN, whereas AHI and ODI quartiles were not. In separate model-specific collinearity analyses using T90 and each hypoxemia-related index as continuous variables, VIFs ranged from 1.12 to 1.26, indicating no evidence of problematic multicollinearity (Supplementary Table E5).

After adjusting for potential confounders detailed in Model 2, the restricted cubic spline analysis showed no statistically significant overall associations or departures from linearity linking SBII, HB, AHI, or ODI with the odds of incident HTN (all *P* for overall association ≥0.229 and all *P* for nonlinearity ≥0.189) (Figure 2 and Supplementary Table E6).

**Figure 2:**
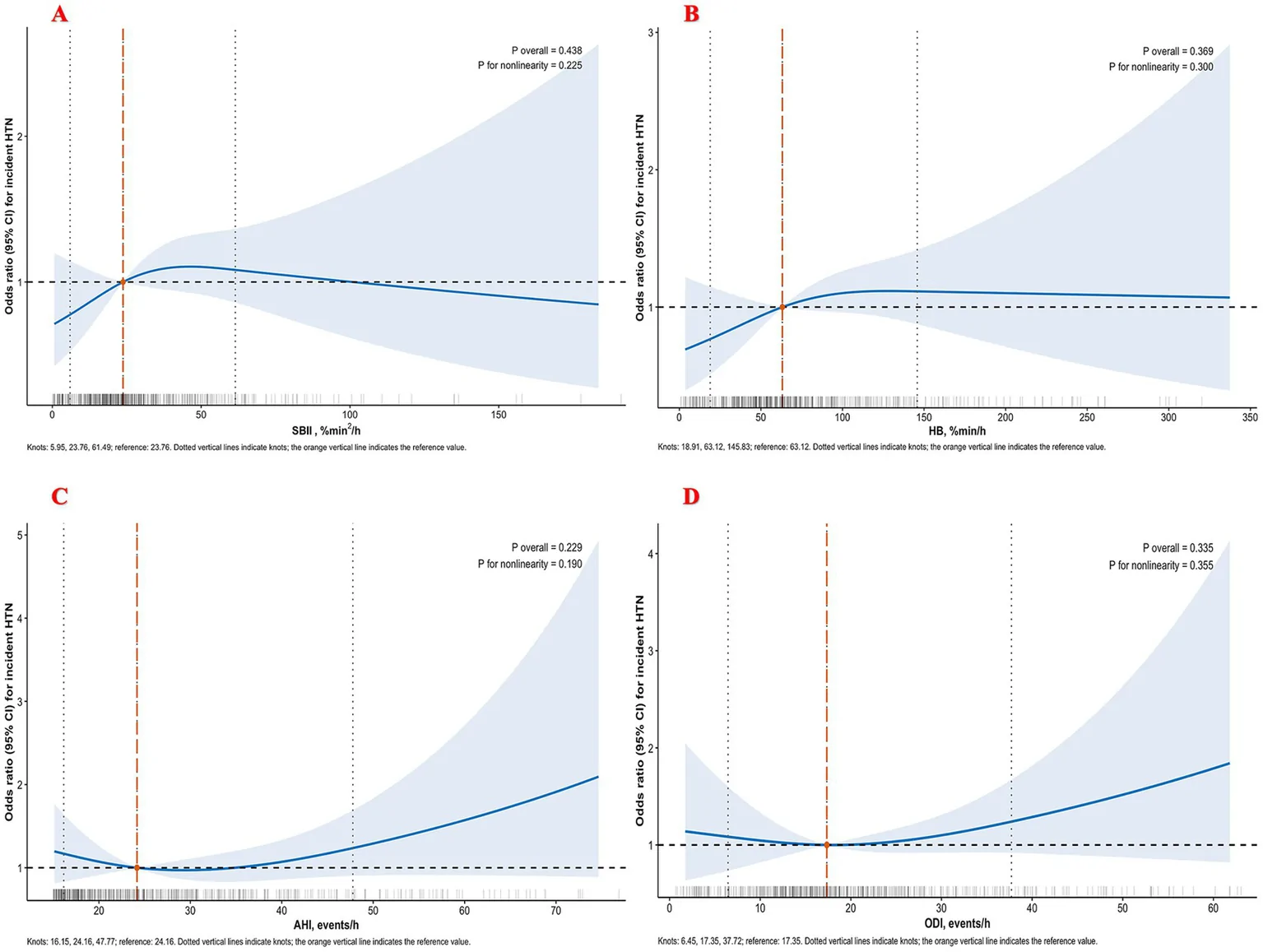
Restricted cubic spline analysis of SBII, HB, AHI, and ODI in relation to incident HTN. **(A)** SBII; **(B)** HB; **(C)** AHI; **(D)** ODI. All indices were assessed as continuous variables using restricted cubic spline analyses. Three knots were placed at the 10th, 50th, and 90th percentiles, with the median used as the reference value (OR = 1). The solid curves represent adjusted ORs, and the shaded areas represent 95% CIs; the horizontal dashed line indicates an OR of 1. The displayed exposure ranges were restricted to the 1st–99th percentiles, whereas all observations were retained in model estimation without truncation or winsorization. Rug marks along the x-axis indicate the distribution of observed exposure values. Models were adjusted for the covariates included in Model 2. Exact knot and reference values observed and displayed ranges, and model sample sizes and event counts are provided in Supplementary Table E6. HTN, hypertension; SBII, sleep breathing impairment index; HB, hypoxic burden; AHI, apnea-hypopnea index; ODI, oxygen desaturation index.

### Discriminatory performance of SBII, HB, AHI and ODI for incident HTN

The clinical base model had a C-statistic of 0.614 (95% CI, 0.564–0.664). After separately adding quartiles of SBII, HB, AHI, and ODI, the C-statistics increased to 0.632 (95% CI, 0.582–0.682), 0.631 (95% CI, 0.580–0.681), 0.630 (95% CI, 0.580–0.680), and 0.624 (95% CI, 0.574–0.674), respectively (Table 4). These increases were small (ΔC, 0.010–0.018) and were not statistically significant compared with the clinical base model (all *p* > 0.05). Moreover, the discriminative performance of the HB-, AHI-, and ODI-augmented models did not differ significantly from that of the SBII-augmented model (all *p* > 0.05).

**Table 4:** Discriminative performance of multivariable models incorporating SBII, HB, AHI, or ODI quartiles for incident HTN.

Model	C-statistic (95% CI)	ΔC vs base model	*p* value vs base model	*p* value vs SBII model
Clinical base model	0.614 (0.564–0.664)	Reference	—	—
Base + SBII quartiles	0.632 (0.582–0.682)	0.018	0.172	Reference
Base + HB quartiles	0.631 (0.580–0.681)	0.017	0.220	0.922
Base + AHI quartiles	0.630 (0.580–0.680)	0.016	0.267	0.957
Base + ODI quartiles	0.624 (0.574–0.674)	0.010	0.342	0.615

The clinical base model included gender, race, age, BMI, smoking status, alcohol use, diabetes mellitus, and chronic obstructive pulmonary disease. Each extended model additionally included one OSA-related index categorized into quartiles, with Q1 as the reference. All models were fitted using the same complete-case sample.

ΔC represents the difference in the C-statistic between each extended model and the clinical base model. *p* values were calculated using paired DeLong tests.

All discrimination analyses were conducted using the same complete-case sample of 557 participants, including 181 incident HTN events.

SBII, sleep breathing impairment index; HB, hypoxic burden; AHI, apnea-hypopnea index; ODI, oxygen desaturation index; HTN, hypertension; CI, confidence interval.

In Bootstrap internal validation with 1,000 resamples, the optimism-corrected C-statistics ranging from 0.506 to 0.512, with calibration intercepts ranging from −0.421 to −0.391 and calibration slopes ranging from 0.422 to 0.462. These findings indicated substantial optimism and limited internally validated predictive performance (Supplementary Table E7).

## Discussion

In this prospective cohort of participants with OSA and without HTN at baseline, those in the highest quartiles of SBII and HB had 71 and 69% higher odds of incident HTN, respectively, than those in the lowest quartiles after multivariable adjustment. In contrast, the corresponding highest quartiles of AHI and ODI were not significantly associated with incident HTN. Adding any of the indices to the clinical base model resulted in only small, nonsignificant improvements in the C-statistic, and the HB-augmented model did not differ significantly from the SBII-augmented model. These findings suggested that SBII and HB may capture aspects of the magnitude and duration of event-related hypoxemia relevant to subsequent HTN that were not fully represented by AHI and ODI, although their incremental discriminative value beyond clinical covariates was limited.

AHI and ODI are commonly used to assess OSA severity, but it ignores event duration and does not represent the degree of oxygen desaturation (10, 17). Previous longitudinal studies that evaluated the relationship between AHI and incident HTN have yielded inconsistent findings. In the Wisconsin Sleep Cohort, Peppard et al. (18) followed 709 participants for 4 years and reported a dose–response association between increasing AHI and the presence of HTN. In the SHHS, O’Connor et al. (19) found that higher estimates of incident HTN with increasing AHI, but the association was substantially attenuated after adjustment for BMI and other covariates. More recently, a multicenter study of 450 participants with OSA aged ≥60 years found that higher AHI was independently associated with new-onset HTN during a median follow-up of 33 months (14). Conversely, a retrospective study found no significant differences in AHI or ODI between patients with and without incident HTN (20). These inconsistent findings may reflect differences in age, obesity, baseline BP, OSA severity, and outcome definitions across study populations. In the present study, neither AHI nor ODI was significantly associated with incident HTN after multivariable adjustment. These findings suggested that conventional event-frequency indices may not adequately capture all dimensions of OSA-related hypoxemic exposure relevant to HTN development.

In contrast to AHI and ODI, HB and SBII provide more integrated characterizations of OSA-related hypoxic stress. HB quantifies the cumulative area under respiratory event-associated desaturation curves, whereas SBII additionally integrates the duration of respiratory events with their associated desaturation areas (8–10). Previous studies have primarily related HB to cross-sectional BP levels, ambulatory BP phenotypes, or treatment-related BP changes. In the Multi-Ethnic Study of Atherosclerosis, Kim et al. (21) found that higher HB was associated with higher systolic and diastolic BP among participants not receiving antihypertensive medications. Messineo et al. (6) subsequently demonstrated that greater treatment-related reductions in HB were associated with larger reductions in systolic BP^23^. Moreover, HB has been associated with incident cardiovascular events and mortality in population-based cohorts (8, 22). Evidence regarding SBII remains more limited: higher SBII has been associated with an elevated estimated odds of atrial fibrillation, cardiovascular disease, and cardiovascular mortality (9, 10, 23), but its association with incident HTN has not been specifically evaluated. In the present study, the highest quartiles of both HB and SBII were associated with higher odds of incident HTN, and no significant difference was observed between their C-statistics. Their comparable performance may reflect their shared quantification of cumulative respiratory event-related hypoxic stress. These findings extend previous BP- and cardiovascular disease-related evidence by linking both integrated hypoxic indices to incident HTN.

Several biological mechanisms may underlie the observed associations of SBII and HB with incident HTN. Recurrent obstructive respiratory events produce intermittent hypoxemia and arousals, stimulating peripheral chemoreflex pathways and sympathetic outflow; the termination of these events is accompanied by abrupt nocturnal BP surges that are closely related to sympathetic activity (2, 24, 25). Repeated hypoxia–reoxygenation may also promote oxidative stress and inflammatory signaling, reduce nitric oxide bioavailability, and impair microvascular endothelial function (2, 25, 26). Over time, persistent sympathetic activation, activation of the renin–angiotensin–aldosterone system, and impaired baroreflex regulation may promote vasoconstriction, sodium retention, arterial stiffness, and vascular remodeling, thereby contributing to sustained HTN (2, 25, 27). HB and SBII integrate multiple dimensions of oxygen desaturation and provide a more comprehensive characterization of OSA-related hypoxic stress (8–10). Compared with AHI and ODI, which primarily quantify the frequency of respiratory events and oxygen desaturations, respectively, these integrated indices may more closely reflect the physiological burden relevant to incident HTN. This mechanistic relevance may partly explain why the highest quartiles of SBII and HB, but not quartiles of AHI or ODI, were associated with incident HTN in the present study.

Although SBII and HB were associated with incident HTN in the primary association analyses, the clinical base model already showed modest discrimination, with a C-statistic of 0.614. Adding quartile-categorized SBII, HB, AHI, or ODI increased the C-statistic only slightly (ΔC, 0.010–0.018), and none of these increases was statistically significant. Furthermore, the discriminative performance of the HB-augmented model did not differ significantly from that of the SBII-augmented model. The similar performance of SBII and HB may partly reflect the substantial overlap in the information captured by these integrated hypoxemia indices, as supported by their strong correlation (*r* = 0.909; *p* < 0.001). Bootstrap internal validation yielded optimism-corrected C-statistics of 0.506–0.512 and calibration slopes of 0.422–0.462, suggesting substantial optimism and limited internally validated predictive performance. The presence of statistically significant associations without meaningful improvement in discrimination is not contradictory, because modest associations confined primarily to the highest exposure quartiles may contribute little to individual-level outcome classification beyond established clinical covariates. Therefore, these findings support the potential relevance of SBII and HB as markers associated with subsequent HTN but do not establish clinically meaningful incremental prediction or their suitability as stand-alone prediction tools. Further studies using larger independent cohorts and prespecified clinical prediction models are needed to evaluate their incremental discrimination, calibration, clinical utility, and external validity (28, 29).

This study has some strengths. Firstly, the community-based prospective design and the exclusion of participants with HTN at baseline enabled the evaluation of newly developed HTN. Secondly, OSA-related indices were derived from standardized overnight PSG, providing objective measurements of sleep-disordered breathing and nocturnal hypoxemia. Thirdly, SBII, HB, AHI, and ODI were directly compared within the same study population. Finally, the combined use of categorical logistic regression, restricted cubic spline analysis, and C-statistic comparisons also enabled complementary evaluation of their associations with incident HTN and discriminative ability.

However, several limitations should be considered. Firstly, the underrepresentation of younger and more diverse racial and ethnic groups limits the generalizability of our findings. Secondly, selection bias cannot be excluded because excluded participants had a higher proportion of males, a lower proportion of White, a lower BMI, higher baseline systolic and diastolic BP, and a higher prevalence of DM than those in the analytic sample. Thirdly, all sleep indices were derived from a single baseline PSG and may therefore be affected by night-to-night variability. Fourthly, SBII and HB primarily quantify event-related hypoxia and may ignore other physiological dimensions of OSA, such as loop gain, arousal threshold, critical closing pressure, and muscle compensation. Fifthly, although participants with HTN at baseline were excluded, baseline BP was not included in the primary models because subthreshold elevations may partly reflect the effects of recurrent OSA-related hypoxemia. Our findings therefore represent overall prospective associations rather than associations independent of baseline BP. Finally, the absence of 24-h ambulatory BP monitoring limited the comprehensive assessment of circadian BP patterns, particularly nocturnal BP and dipping status.

## Conclusion

In conclusion, among participants with OSA and without HTN at baseline, exploratory quartile-based analyses indicated that the highest quartiles of SBII and HB were associated with incident HTN after multivariable adjustment, whereas the corresponding quartiles of AHI and ODI were not statistically significant. By capturing cumulative characteristics of event-related hypoxemia that are not reflected by event frequence alone, SBII and HB may be relevant to incident HTN. However, the absence of significant associations in the continuous and overall spline analyses, together with the limited incremental discrimination beyond clinical covariates, warrants cautious interpretation. Further studies are needed to establish clinically interpretable thresholds and evaluate their external validity and clinical utility.

## Data Availability

Publicly available datasets were analyzed in this study. This data can be found here: The datasets analyzed in this study are available from the National Sleep Research Resource (NSRR), Sleep Heart Health Study repository: https://sleepdata.org/datasets/shhs. Access is subject to registration and approval of a data access request.

## Footnotes

1
http://www.rproject.org
