# Supplementary material for: Conventional and novel hypoxemia-related indices for obstructive sleep apnea severity and incident hypertension: a prospective cohort study
Source: Front Neurol. 2026 Oct 7;17:1973505. doi: 10.3389/fneur.2026.1973505 (PMC13683184; doi:10.3389/fneur.2026.1973505)
Supplement: Supplementary file 1 [file Supplementary_file_1.docx]

**Supplementary Materials**

**Figure E1.** **Pearson correlations among SBII, HB, AHI, and ODI.**

Correlations between the OSA severity indices were evaluated using Pearson correlation coefficients. The values shown represent correlation coefficients, with the color scale indicating the direction and magnitude of the correlations.

Abbreviations: SBII, sleep breathing impairment index; HB, hypoxic burden; AHI, apnea-hypopnea index; ODI, oxygen desaturation index.

**Details of missing data**

Missing data were present for smoking status in 1 participant (0.17%), alcohol use in 40 (6.66%), diabetes mellitus in 17 (2.83%), and COPD in 4 (0.67%); age category, race, gender, and BMI category were complete. Overall, 44 participants had missing data for at least one variable required in the multivariable models, leaving 557 participants in the common complete-case sample. The same complete-case sample was used for the multivariable models comparing SBII, HB, AHI, and ODI. For descriptive analyses, percentages were calculated using the number of participants with available data for the corresponding variable as the denominator (**Table E1**).

**Table E1. Availability and missingness of covariate data among the 601 eligible participants.**

| **Covariate** | **Available, n** | **Missing, n (%)** |
| --- | --- | --- |
| Race | 601 | 0 (0.00%) |
| Gender | 601 | 0 (0.00%) |
| Age group | 601 | 0 (0.00%) |
| BMI group | 601 | 0 (0.00%) |
| Smoking status | 600 | 1 (0.17%) |
| Alcohol use | 561 | 40 (6.66%) |
| DM | 584 | 17 (2.83%) |
| COPD | 597 | 4 (0.67%) |

Abbreviations: BMI, body mass index; DM, diabetes mellitus; COPD, chronic obstructive pulmonary disease.

**Table E2. Baseline characteristics between analytic samples and excluded samples.**

| **Variable** | **Excluded sample**  **(N=5203)** | **Analytic sample**  **(N=601)** | ***P*** |
| --- | --- | --- | --- |
| Age, years | 63.00 (55.00, 72.00) | 62.00 (56.00, 70.00) | 0.935 |
| Age group |  |  | 0.142 |
| <60 years (n=221) | 2079 (39.96) | 221 (36.77) |  |
| ⩾60 years (n=380) | 3124 (60.04) | 380 (63.23) |  |
| male, n (%) | 2831 (54.41) | 208 (34.61) | <0.001 |
| BMI, kg/m^2^ | 27.30 (24.51, 30.59) | 29.21 (26.26, 31.98) | <0.001 |
| BMI group |  |  | <0.001 |
| <30 kg/m^2^ (n=352) | 3681 (71.34) | 352 (58.57) |  |
| 30~35kg/m^2^ (n=179) | 1014 (19.65) | 179 (29.78) |  |
| ⩾35kg/m^2^ (n=70) | 465 (9.01) | 70 (11.65) |  |
| Race, n (%) |  |  | <0.001 |
| White | 4359 (83.78) | 548 (91.18) |  |
| African American | 496 (9.53) | 19 (3.16) |  |
| Other | 348 (6.69) | 34 (5.66) |  |
| Alcohol use, n (%) | 2130 (44.12) | 288 (51.34) | 0.001 |
| Current smoking, n (%) | 508 (9.84) | 52 (8.67) | 0.004 |
| Average systolic BP, mm Hg | 126.00 (114.00, 140.00) | 118.00 (110.00, 127.00) | <0.001 |
| Average diastolic BP, mm Hg | 74.00 (66.00, 82.00) | 70.00 (64.00, 76.50) | <0.001 |
| DM, n (%) | 380 (7.68) | 25 (4.28) | 0.004 |
| COPD, n (%) | 59 (1.15) | 3 (0.50) | 0.072 |
| Antidepressants usage, n (%) | 394 (7.58) | 26 (4.35) | 0.005 |
| Benzodiazepines usage, n (%) | 290 (5.59) | 18 (3.01) | 0.010 |
| AHI, events/h | 8.20 (3.55, 16.05) | 24.05 (18.15, 33.72) | <0.001 |
| ODI, events/h | 11.04 (5.04, 30.00) | 17.46 (12.43, 25.37) | <0.001 |
| SBII, %min2/h | 8.84 (3.46, 20.52) | 23.95 (15.16, 39.77) | <0.001 |
| HB, %min/h | 27.78 (12.26, 59.42) | 62.90 (40.67, 96.33) | <0.001 |
| T90, % | 0.13 (0.00, 1.39) | 1.70 (0.37, 5.47) | <0.001 |

Note: Values were presented as median (interquartile range) or number (percentage).

Abbreviations: HTN, hypertension; BMI, body mass index; SBP, systolic blood pressure; DBP, diastolic blood pressure; DM, diabetes mellitus; COPD, chronic obstructive pulmonary disease; SBII, sleep breathing impairment index; HB, hypoxic burden; AHI, apnea-hypopnea index; ODI, oxygen desaturation index; T90, the percentage of sleep time spent with oxygen saturation of <90%.

**Table E3.** **Proportion of incident HTN in each group of SBII, HB, AHI, and ODI.**

| **Variable** | **Q1** | **Q2** | **Q3** | **Q4** | **Global *P* value** | ***P* for trend** |
| --- | --- | --- | --- | --- | --- | --- |
| SBII quartiles, n/N (%) | 42/151 (27.81) | 49/149 (32.89) | 45/151 (29.80) | 58/150 (38.66) | 0.201 | 0.084 |
| HB quartiles, n/N (%) | 43/150 (28.67) | 43/151 (28.48) | 47/150 (31.33) | 61/150 (40.67) | 0.079 | 0.023 |
| AHI quartiles, n/N (%) | 42/150 (28.00) | 52/151 (34.44) | 38/150 (25.33) | 62/150 (41.33) | 0.015 | 0.071 |
| ODI quartiles, n/N (%) | 55/150 (36.67) | 41/150 (27.33) | 43/151 (28.48) | 55/150 (36.67) | 0.150 | 0.948 |

Note: Data were presented as percentages, where n was the number of participants who developed HTN and N is the total number of participants within the corresponding quartile.

Global *P* values compare incident HTN proportions across the four quartiles using Pearson’s chi-square test or Fisher’s exact test, as appropriate. *P* values for trend were calculated using a chi-square test for trend in proportions, with quartiles assigned ordinal scores from 1 to 4.

Abbreviations: HTN, hypertension; SBII, sleep breathing impairment index; HB, hypoxic burden; ODI, oxygen desaturation index; AHI, apnea-hypopnea index.

**Table E4.** **Sensitivity analysis of the associations of SBII, HB, AHI, and ODI with incident HTN after additional adjustment for T90.**

| **Variables** | **Additional Model**  **OR (95%CI)** | ***P*** |
| --- | --- | --- |
| **SBII, %min^2^/h** |  |  |
| **Per 1-SD increase** | 1.05 (0.86–1.27) | 0.652 |
| Q1 (<15.16) | 1.00 | — |
| Q2 (15.16~23.95) | 1.32 (0.78–2.23) | 0.307 |
| Q3 (23.95~39.77) | 1.10 (0.64–1.91) | 0.722 |
| Q4 (>39.77) | 1.80 (1.04–3.12) | 0.036 |
| **HB, %min/h** |  |  |
| **Per 1-SD increase** | 1.11 (0.91–1.35) | 0.295 |
| Q1 (<40.66) | 1.00 | — |
| Q2 (40.66~62.90) | 0.98 (0.57–1.68) | 0.939 |
| Q3 (62.90~96.33) | 1.09 (0.64–1.85) | 0.762 |
| Q4 (>96.33) | 1.80 (1.05–3.10) | 0.034 |
| **AHI, events/h** |  |  |
| **Per 1-SD increase** | 1.13 (0.93–1.39) | 0.218 |
| Q1 (<18.15) | 1.00 | — |
| Q2 (18.15~24.05) | 1.33 (0.79–2.25) | 0.276 |
| Q3 (24.05~33.72) | 0.83 (0.48–1.42) | 0.494 |
| Q4 (>33.72) | 1.56 (0.90–2.71) | 0.116 |
| **ODI, events/h** |  |  |
| **Per 1-SD increase** | 1.13 (0.93–1.37) | 0.223 |
| Q1 (<12.43) | 1.00 | — |
| Q2 (12.43~17.46) | 0.79 (0.47–1.32) | 0.368 |
| Q3 (17.46~25.37) | 0.73 (0.43–1.22) | 0.232 |
| Q4 (>25.37) | 1.06 (0.63–1.79) | 0.820 |

Note: This additional adjusted Model included T90 as a binary covariate (<5% vs ≥5%), plus all covariates included in Model 2. Q1 was the reference.

Abbreviations: HTN, hypertension; SBII, sleep breathing impairment index; HB, hypoxic burden; AHI, apnea-hypopnea index; ODI, oxygen desaturation index; T90, the percentage of sleep time spent with oxygen saturation of <90%; OR, odds ratio; CI, confidence interval.

**Table E5. Collinearity diagnostics for T90 and the hypoxemia-related indices.**

| **Hypoxemia-related index**  **included with T90** | **VIF for the**  **Hypoxemia-related index** | **VIF for T90** |
| --- | --- | --- |
| SBII＋T90 | 1.16 | 1.23 |
| HB＋T90 | 1.16 | 1.22 |
| AHI＋T90 | 1.21 | 1.26 |
| ODI＋T90 | 1.12 | 1.17 |

Note: For the collinearity analyses, both the hypoxemia-related index and T90 were entered as continuous variables, together with the covariates included in Model 2. Only the VIFs for the corresponding index and T90 were presented. VIF values <5 were considered to indicate no problematic multicollinearity.

Abbreviations: SBII, sleep breathing impairment index; HB, hypoxic burden; AHI, apnea-hypopnea index; ODI, oxygen desaturation index; T90, the percentage of sleep time spent with oxygen saturation of <90%; VIF, variance inflation factor.

**Table E6. Restricted cubic spline model specifications and association tests for incident HTN.**

| **Variable** | **Knot locations**  **(P10, P50, P90)** | **Reference value (OR=1)** | **Observed range** | **Displayed range**  **(P1–P99)** | ***P* overall** | ***P* for nonlinearity** | **Model sample, N (events)** |
| --- | --- | --- | --- | --- | --- | --- | --- |
| SBII, %min²/h | 5.95, 23.76, 61.49 | 23.76 | 0.19–243.51 | 0.67–183.54 | 0.438 | 0.225 | 557 (181) |
| HB, %min/h | 18.91, 63.12, 145.83 | 63.12 | 0.82–409.35 | 3.60–337.40 | 0.369 | 0.300 | 557 (181) |
| AHI, events/h | 16.15, 24.16, 47.77 | 24.16 | 15.00–106.15 | 15.12–74.65 | 0.229 | 0.189 | 557 (181) |
| ODI, events/h | 6.45, 17.35, 37.72 | 17.35 | 0.69–102.91 | 1.73–61.81 | 0.335 | 0.355 | 557 (181) |

Note: Knots were placed at the 10th, 50th, and 90th percentiles of each exposure distribution. The median value (50th percentile) was used as the reference (OR = 1).

All models were adjusted for the covariates included in Model 2 and were fitted using the same complete-case sample. All observations were retained in model fitting, and no exposure values were excluded, truncated, or winsorized. Model sample, N (events), denotes the number of participants and incident HTN events included in each model.

Abbreviations: HTN, hypertension; SBII, sleep breathing impairment index; HB, hypoxic burden; AHI, apnea-hypopnea index; ODI, oxygen desaturation index; OR, odds ratio.

**Table E7. Bootstrap internal validation and calibration of the clinical base and index-augmented models for incident HTN.**

| **Model** | **Optimism-corrected C-statistic** | **Calibration intercept** | **Calibration slope** |
| --- | --- | --- | --- |
| Clinical base model | 0.508 | −0.391 | 0.462 |
| Base + SBII quartiles | 0.512 | −0.405 | 0.44 |
| Base + HB quartiles | 0.512 | −0.398 | 0.453 |
| Base + AHI quartiles | 0.509 | −0.400 | 0.447 |
| Base + ODI quartiles | 0.506 | −0.421 | 0.422 |

Note: Internal validation was performed using 1000 bootstrap resamples. An intercept calibration of 0 and a slope calibration of 1 indicated ideal calibration.

Abbreviations: HTN, hypertension; SBII, sleep breathing impairment index; HB, hypoxic burden; ODI, oxygen desaturation index; AHI, apnea-hypopnea index.

**SBII Algorithm**

Manually scored apnea and hypopnea events were used as airflow events. For each event, the algorithm searched for associated oxygen desaturations within a 100-s window beginning at event onset. The area of each desaturation was quantified by integrating the instantaneous reduction in SpO₂ from the saturation level at desaturation onset. Oximetry artifacts, defined by default as SpO₂ values <20%, were corrected using adjacent valid measurements. Only respiratory events accompanied by a maximum SpO₂ reduction ≥3% were included. For each eligible event, the respiratory event duration was multiplied by its cumulative desaturation area. SBII was obtained by summing these event-specific values and normalizing them to total sleep time:

$SBII= {\sum_{\begin{aligned} h\in L_{f} \\ f\in F \end{aligned}} \left( T_{\left( f, duration \right)}*\int_{t_{\left( l, start \right)}}^{t_{\left( l, stop \right)}} \left( S_{t_{\left( h, start \right)}}-S_{t} \right)dt \right)}/{T_{totalSleepTime}}$

In the provided equations, "$F$" denotes the set of airflow events, "$L_{f}$" represents the desaturations detected within the 100-s search window for event. "$T_{\left( f, duration \right)}$" is the duration of the airflow event. "$t_{\left( l, start \right)}$" and "$t_{\left( l, stop \right)}$" indicate the beginning and end of the corresponding desaturation. "$S_{t}$" is the SpO_2_ value at time "t", and "$S_{t_{\left( l, start \right)}}$" is the saturation level at desaturation onset. "$\left( S_{t_{\left( l, start \right)}}-S_{t} \right)$" indicates the depth of oxygen desaturation at the precise time point "t".The integral represents the cumulative desaturation area. The SBII algorithm was developed in our previous studies,^1, 2^ and detailed instructions and an automated calculation platform are publicly available at <http://61.160.194.165:3080/SBII/>.

**Reference (included in the reference list of manuscript)**

1.	Hui X, Cao W, Xu Z, Guo J, Luo J, Xiao Y. Hypoxic indices for obstructive sleep apnoea severity and cardiovascular disease risk prediction: A comparison and application in a community population. *Respirology*. 2024;29:825-834

2.	Wang L, Hui X, Cao W, Huang R, Xiao Y. Sleep breathing impairment index predicts incident atrial fibrillation in obstructive sleep apnea: A prospective cohort study. *Sleep Med*. 2025;129:386-393
